# Supplementary figures and images for: CXCR4 and MIF are required for neutrophil extracellular trap release triggered by Plasmodium-infected erythrocytes
Source: PLoS Pathog. 2020 Aug 14;16(8):e1008230. doi: 10.1371/journal.ppat.1008230 (PMC7449500; doi:10.1371/journal.ppat.1008230)

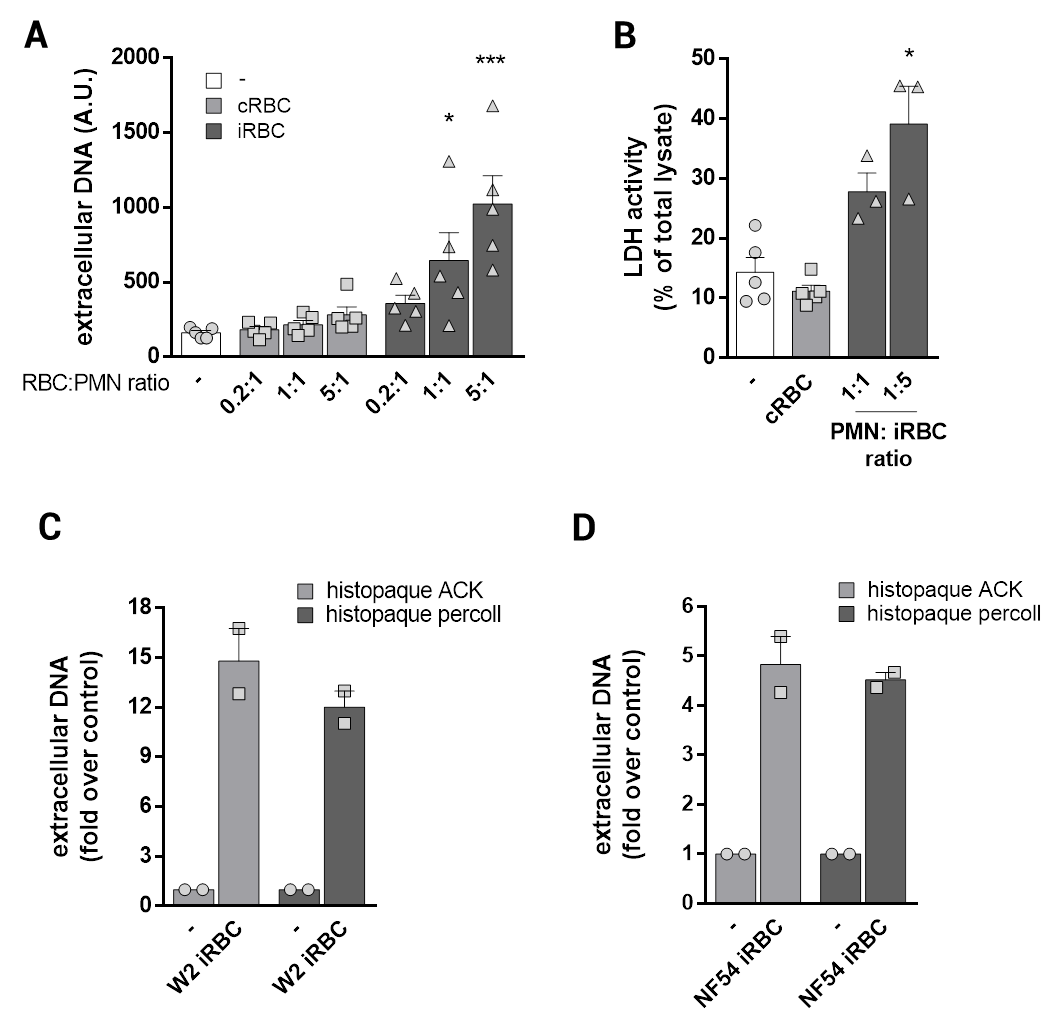

Supplement: S1 Fig — (A) Fluorimetric determination of NET production by human neutrophils in the presence of P. falciparum-infected red blood cells (iRBC) or uninfected RBC (cRBC) at varying red blood cell:neutrophil ratios and represented as means ± S.E.M. of the extracellular DNA fluorescence signal (in arbitrary units). (B) Determination of lactate dehydrogenase (LDH) activity in culture supernatants of human neutrophils incubated with infected red blood cells (iRBC) at two different neutrophil:red blood cell ratios for 3 hours. Uninfected red blood cells (cRBC) were used as controls. LDH activity in culture supernatants was compared to the total intracellular LDH activity as determined in neutrophil cell lysates. (C) Human neutrophils were purified by two different protocols: histopaque followed by ACK lysis of red blood cells (light grey bars) or histopaque followed by percoll gradient (dark grey bars). NET release was determined in response to erythrocytes infected with P. falciparum W2 strain. (D) NET release by human neutrophils purified using different protocols in response to erythrocytes infected with P. falciparum NF54 strain. * P< 0.05 and *** P< 0.001 relative to unstimulated neutrophils. (TIF) [file ppat.1008230.s001.tif]

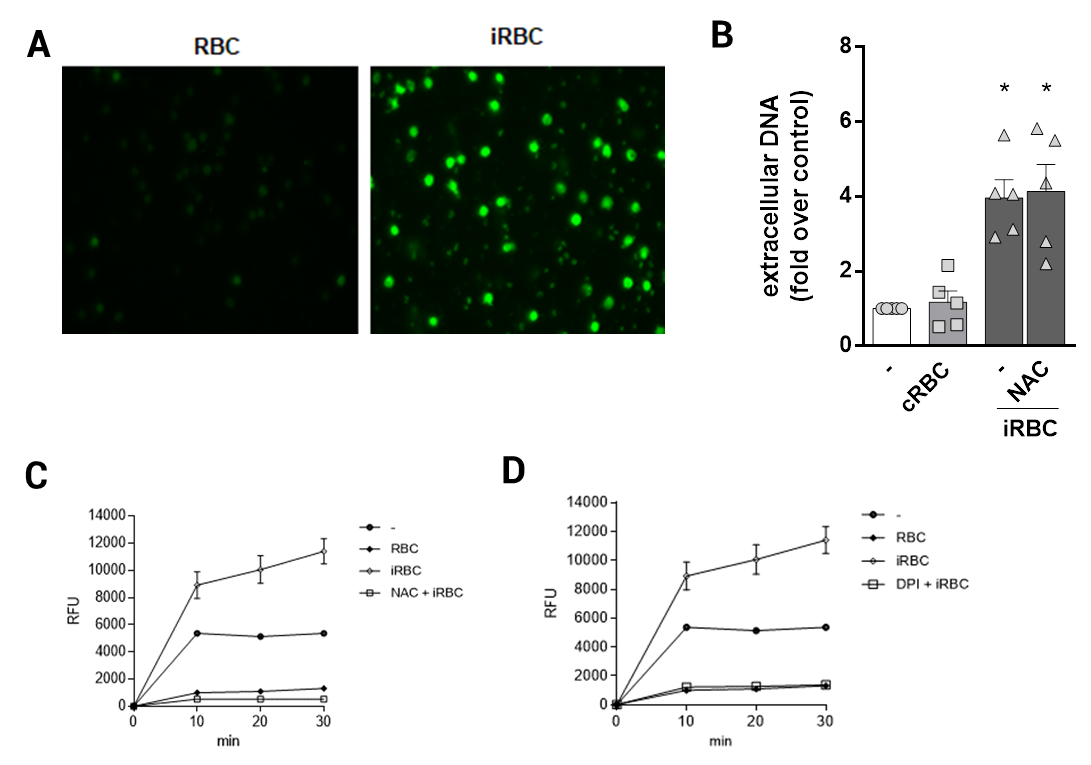

Supplement: S2 Fig — (A) Representative fluorescence images of ROS production by human neutrophils incubated with infected (iRBC) or uninfected red blood cells (RBC) at a 1:5 ratio in the presence of the ROS-sensitive CM-H2DCFDA probe. (B) Human neutrophils were treated with NAC (10 μM) for 30 minutes and then incubated with P. falciparum-infected red blood cells (iRBC). NET production was determined by fluorimetry. Uninfected red blood cells (cRBC) were used as control. Data are presented as means ± S.E.M. of the fold induction of extracellular DNA signal relative to resting neutrophils. (C and D) Kinetics of ROS production by human neutrophils incubated with infected red blood cells (iRBC) and treated or not with antioxidants DPI (C) or NAC (D). ROS production was evaluated by fluorimetry every 10 minutes for 30 minutes in the presence of CM-H2DCFDA. (TIF) [file ppat.1008230.s002.tif]

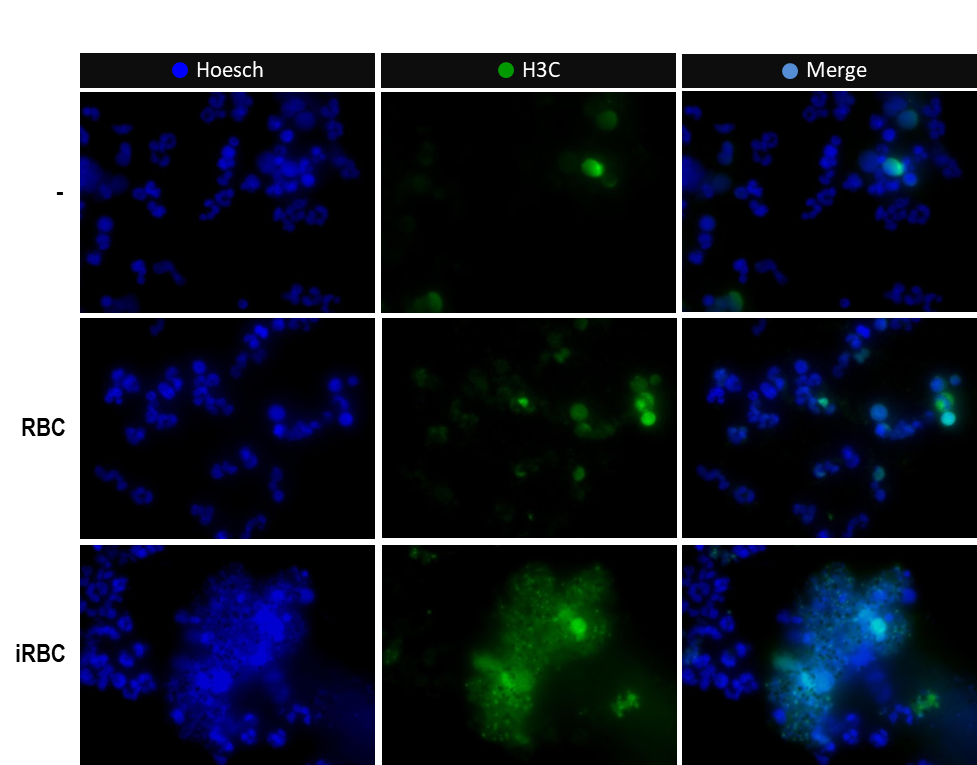

Supplement: S3 Fig — Representative immunofluorescence images of human neutrophils incubated with P. falciparum-infected (iRBC) or uninfected (cRBC) red blood cells at a 1:5 ratio for 3 hours and stained for DNA (blue) and citrullinated histone H3 (green). Unstimulated neutrophils were used as controls. (TIF) [file ppat.1008230.s003.tif]

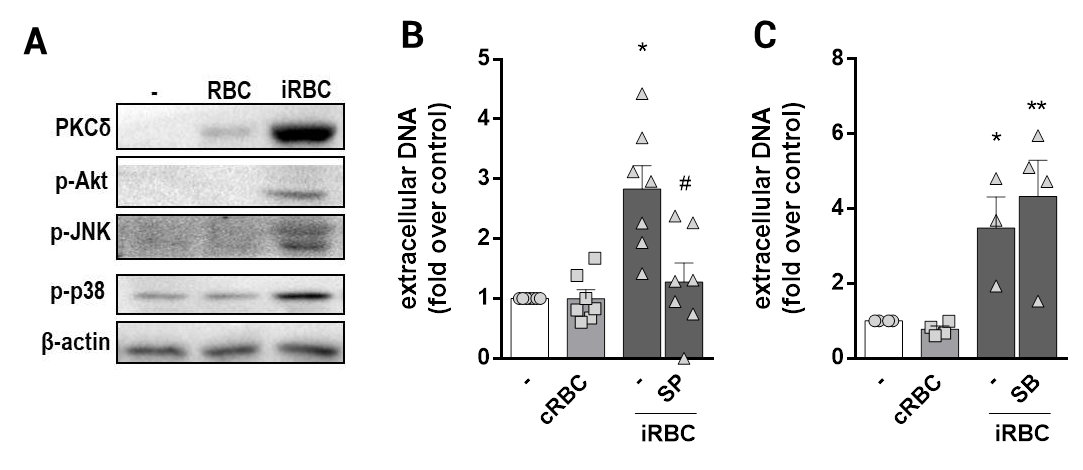

Supplement: S4 Fig — (A) Representative westernblot images of total cell extracts of neutrophils incubated with infected (iRBC) or uninfected (cRBC) red blood cells at a 1:5 ratio. Westernblot was used for the detection of total PKCδ and phosphorylated Akt (p-Akt), JNK (p-JNK) and p38 (p-p38). β-actin was used as loading control. Unstimulated neutrophils were used as controls. (B and C) Human neutrophils were treated with SB239063 (SB, 20 μM) (B) or SP600125 (SP, 40 μM) (C) for 30 minutes and then incubated with P. falciparum-infected red blood cells (iRBC) at a 1:5 ratio for 3 hours. NET production was determined by fluorimetry. Uninfected red blood cells (cRBC) were used as control. Data are presented as means ± S.E.M. of the fold induction of extracellular DNA signal relative to resting neutrophils. * P< 0.05 and ** P< 0.01 relative to controls incubated with cRBC, # P< 0.01 relative to untreated control. (TIF) [file ppat.1008230.s004.tif]

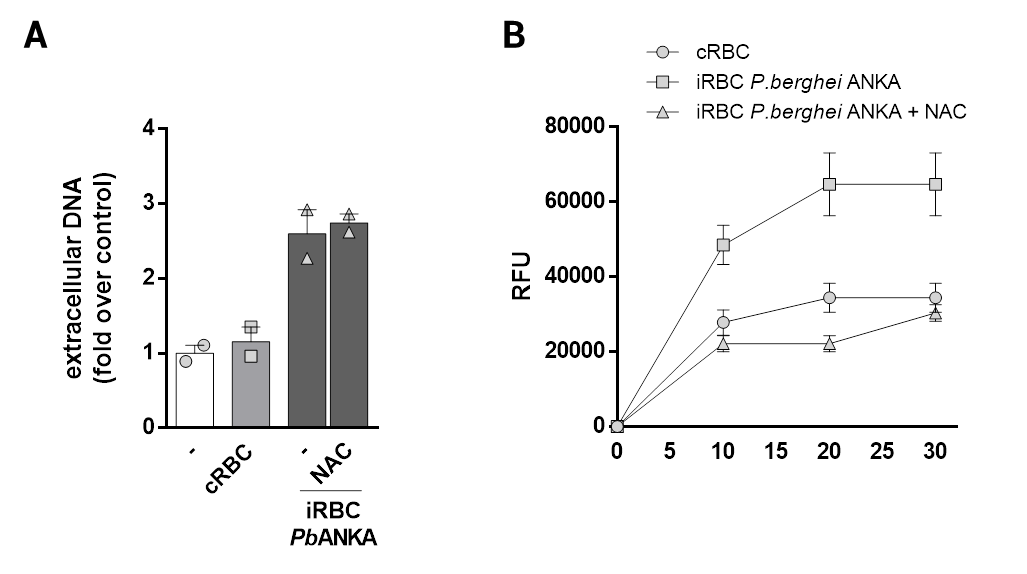

Supplement: S5 Fig — (A) Murine neutrophils were treated with NAC (10 μM) for 30 minutes and then incubated with P. berguei ANKA-infected red blood cells (iRBC). NET production was determined by fluorimetry. Uninfected red blood cells (cRBC) were used as control. Data are presented as means ± S.E.M. of the fold induction of extracellular DNA signal relative to resting neutrophils. (B) Kinetics of ROS production by murine neutrophils incubated with infected red blood cells (iRBC) and treated or not with NAC. ROS production was evaluated by fluorimetry every 10 minutes for 30 minutes in the presence of CM-H2DCFDA. (TIF) [file ppat.1008230.s005.tif]

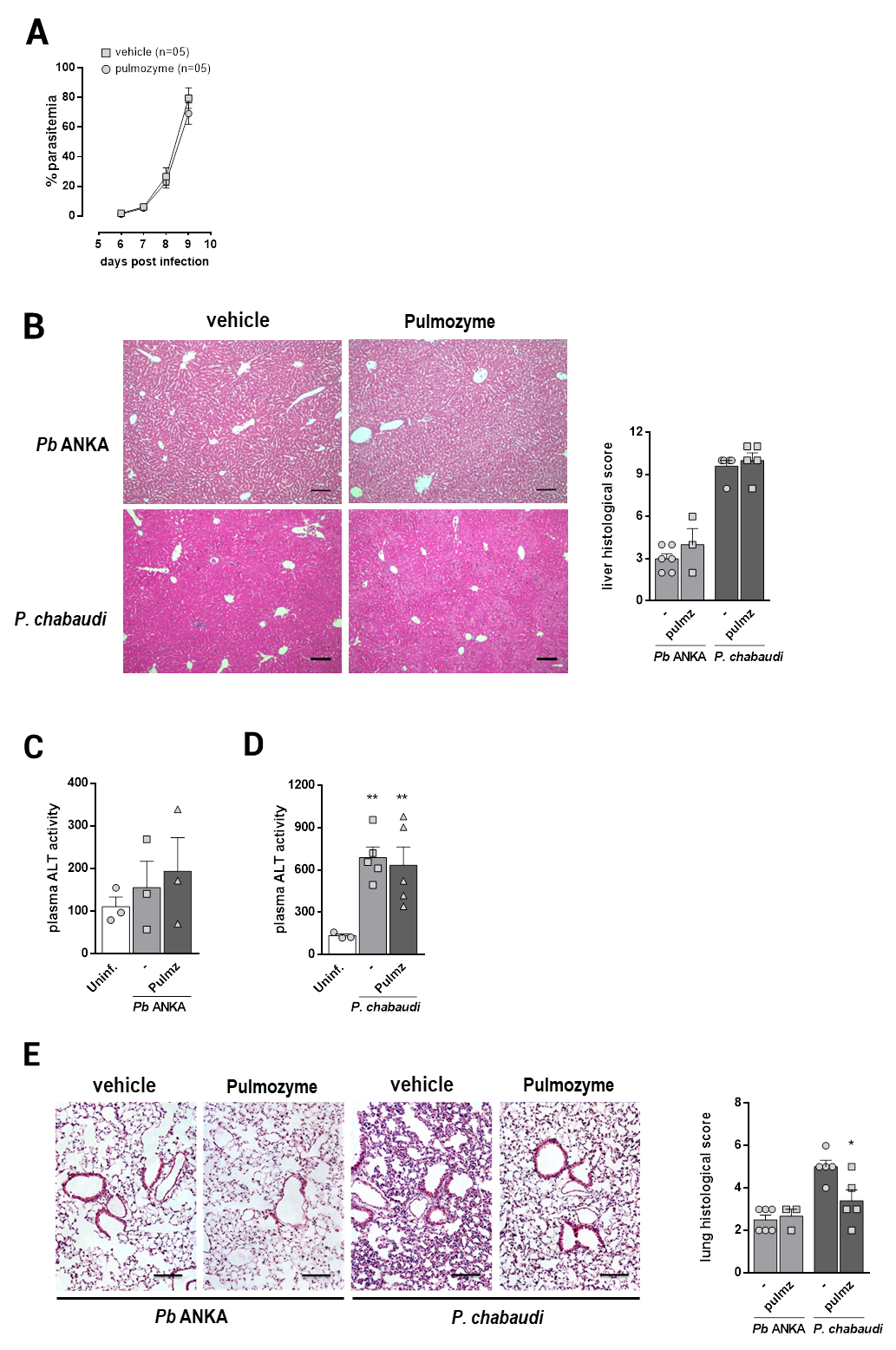

Supplement: S6 Fig — (A) Parasitemia in mice infected with P. chabaudi and treated or not with DNAse (pulmozyme). (B) Histological analysis of liver samples from P. berguei ANKA or P. chabaudi infected C57/B6 mice, treated or not with DNAse (pulmozyme). Samples were collected at day 6 post infection. Representative images (50μm scale bar) and histological scores of 3 to 6 mice in each group. (C and D) ALT levels in plasma of mice infected with P. berguei ANKA (C) or P. chabaudi (D) treated or not with DNAse (pulmz). (E) Histological analysis of lung samples from P. berguei ANKA or P. chabaudi infected C57/B6 mice, treated or not with DNAse (pulmozyme). Samples were collected at day 6 post infection. Representative images (50μm scale bar) and histological scores of 3 to 6 mice in each group. Data are presented as means ± S.E.M. * P< 0.05 and ** P< 0.01 relative to uninfected controls. (TIF) [file ppat.1008230.s006.tif]
